# The Efficacy and Safety of Transcatheter Arterial Embolization to Treat Renal Hemorrhage after Percutaneous Nephrolithotomy

**DOI:** 10.1155/2019/6265183

**Published:** 2019-04-22

**Authors:** Nan Du, Jing-Qin Ma, Jian-Jun Luo, Qing-Xin Liu, Zi-Han Zhang, Min-Jie Yang, Tian-Zhu Yu, Yun Tao, Rong Liu, Wen Zhang, Zhi-Ping Yan

**Affiliations:** ^1^Department of Interventional Radiology, Zhongshan Hospital, Fudan University, Shanghai 200032, China; ^2^Shanghai Institute of Medical Imaging, Shanghai 200041, China

## Abstract

**Purpose:**

The aim of this study was to evaluate the safety and efficacy of transcatheter arterial embolization (TAE) in patients with renal hemorrhage after percutaneous nephrolithotomy (PCNL) and evaluate the risk factors that may result in severe bleeding requiring TAE.

**Methods:**

We retrospectively reviewed 121 patients with post-PCNL renal hemorrhage. Thirty-two patients receiving endovascular embolization were compared with 89 patients only receiving conservative treatment. The demographic and clinical data were recorded and compared between the two groups. The values of estimated glomerular filtration rate (eGFR) and serum creatinine (SCr) were recorded preoperatively, postoperatively, and at last follow-up and analyzed to evaluate the safety and efficiency of TAE.

**Results:**

The successful hemostasis rate of conservative therapy was 73.6% (89/121) and that of TAE was 100% (32/32). SCr and eGFR were not significantly different before PCNL and after the last follow-up of TAE (SCr: 0.95 vs. 0.95 mg/dl,* P*=0.857; eGFR: 86.77 vs. 86.18 ml/min/1.73m^2^,* P*=0.715). The univariate analysis demonstrated that advanced age, urinary tract infection, and diabetes mellitus were significantly associated with severe bleeding during PCNL. Multivariate analysis further identified that diabetes mellitus was an independent risk factor for severe bleeding needing TAE [odds ratio (OR): 3.778, 95% confidence interval (CI):1.276-11.190, and* P*=0.016].

**Conclusion:**

TAE is a safe and effective procedure to treat renal hemorrhage that cannot be resisted by conservative treatment after PCNL. Diabetes mellitus was associated with high risks of severe bleeding needing TAE after PCNL.

## 1. Introduction

Percutaneous nephrolithotomy (PCNL) is a safe and effective treatment for patients with upper urinary calculi. Hematuria is a major and life-threatening complication of PCNL which requires prompt management [1–3]. Previous study indicated that transfusion rates vary from 1% to 11% and embolization rates vary from 0.6% to 2.6% after PCNL [4–6]. Before embolization, angiography was often performed for diagnosis of iatrogenic renal arterial lesions [2, 7, 8]. Moreover, transcatheter arterial embolization (TAE) has been regarded as an effective method for postoperative hemorrhage of PCNL [7, 9]. Before receiving interventional therapy, patients often undergo conservative treatment to hemostasis, including nephrostomy tube clamping, adequate hydration, hemostatic drug using, and blood transfusion [10–12]. Patients with hemodynamic instability or refractory bleeding who cannot achieve hemostasis by conservative therapy have been recommended to angiographic embolization for diagnosis and treatment [11, 13]. Although the efficacy of TAE for post-PCNL hemorrhage has been previously explored [11, 14], the long-term effects on renal function remain to be further confirmed. However, existing studies have not directly compared patients undergoing TAE with patients only receiving conservative treatment. The purpose of this study was to evaluate the safety and efficacy of TAE for patients with post-PCNL severe hemorrhage. Furthermore, we compared patients receiving TAE with patients treated conservatively to explore the risk factors associated with severe hemorrhage.

## 2. Methods

### 2.1. Patients Selection

From October 2010 to June 2017, we retrospectively reviewed 812 patients who underwent PCNL procedures. A total of 121 patients (14.9%), including 89 cases who received conservative treatment and 32 cases who received TAE, were enrolled in this study. The indications for angiography and embolization in our hospital included signs indicative of vascular injury (such as hemodynamic instability requiring blood transfusion, nonresolving hematuria in hemodynamically stable patients, and nonremitting flank pain); uncontrolled intraoperative blood loss; and imaging evidence of vascular injury (pseudoaneurysm formation, active extravasation of contrast medium, creation of arteriovenous fistula, etc.). This study was approved by the ethics committee and institutional review board of our institution. All patients signed informed consent before interventional angiography and embolization.

### 2.2. Data Collection

Demographic and clinical details recorded included age, sex, body mass index (BMI), stone size, side and location, medical comorbidities, and history of antiplatelet therapy and anticoagulation therapy. Types of lesions on angiography were classified into contrast medium extravasation, pseudoaneurysm (PA), arteriovenous fistula (AVF), arteriocalyceal fistula (ACF), or a complex lesion. The levels of hemoglobin (HB) were recorded at preoperative PCNL, postoperative PCNL, and before the day of TAE. Blood loss was defined as HB drop between preoperative PCNL and the day before conservative treatment/TAE. Other laboratory data accessed included platelet count, C-reactive protein (CRP), procalcitonin, urine white blood cell count, and the levels of eGFR and SCr before TAE, after embolization, and at last follow-up. Urinary tract infection was diagnosed by abnormal laboratory data of the leukocyte count in blood and urine, the level of neutrophil, and CRP.

The TAE success and complications were independently evaluated. TAE success was defined as the absence of clinical and imaging evidence of further bleeding within 1 week and stable renal function. Imaging examination, such as ultrasonography and computed tomography (CT) imaging, was performed during the follow-up time. Days from post-PCNL hemorrhage to discharge were recorded as hospitalization days and compared between the two groups. Whether patients received transfusion during hospitalization and the units of transfusion were recorded.

### 2.3. PCNL Procedures

All patients underwent PCNL in the guidance of fluoroscopy. Percutaneous access to the calyx was achieved under general anesthesia. After the tract was dilated with nephrostomy dilator set, an 18-Fr Amplatz sheath (Cook, Inc., Bloomington, USA) was inserted into the targeted calyx. Nephroscopy was performed with a Wolf Nephroscope, and the stones were fragmented using a pneumatic lithotripter or holmium laser. The stone fragments were removed with irrigation or grasping forceps. At the end of the procedure, a ureteral stent and a nephrostomy tube were left to ensure postoperatively adequate urine drainage. If there was no complication or significant residual stone, the nephrostomy tube was removed after 3 to 5 days, and the nephrostomy tube was removed after 2 to 4 weeks. Patients were closely monitored to exclude bleeding. According to the extent of bleeding, patients received conservative therapy or further TAE.

### 2.4. TAE Procedures

Transfemoral catheterization was performed under local anesthesia. After vascular access was obtained, a 4- or 5-French pigtail catheter (Cook, Inc., Bloomington, IN) was placed in the abdominal aorta. Digital subtraction angiography (DSA) using Visipaque (GE Healthcare, Cork, Ireland) was performed to evaluate the renal arteries, accessory renal arteries, lumbar arteries, and other collateral vessels. Then, angiography with a 4- or 5-Fr Cobra catheter (Cook, Inc.) was performed to evaluate the renal artery conditions and the location of the lesions. A 2.8-Fr Renegade catheter (Boston Scientific, Natick, MA) was used for superselective embolization of the culprit artery as distally as possible to minimize the loss of normal renal parenchyma. Embolization materials such as coils (Cook, Inc.), gelatin sponge (Jingling, Jiangsu, China), and* n*-butyl-2-cyanoacrylate (NBCA) (Compont, Beijing, China) were used according to the types of the lesions. After embolization, angiogram was performed to verify the occlusion of the culprit arteries and to assess the percent of parenchymal loss (infarction area). All the patients were carefully monitored after the procedure to exclude potential rebleeding.

## 3. Statistical Analysis

Statistical analysis was performed using SPSS 22.0 (SPSS, Chicago, Illinois). Categorical variables were presented as numbers with percentage, and continuous variables were presented as mean ± standard deviation (SD). The differences between the groups were evaluated using the Chi-square test or Fisher's exact test for categorical variables and the Student* t*-test or Mann-Whitney U test for continuous variables. Multivariate binary logistic regression was used for multivariate analysis. Odds ratio (OR) with 95% confidence interval (CI) was used to measure the magnitude of association. A two-sided* P* value <0.05 was considered to be statistically significant.

## 4. Results

In the present study, 3.9% (32 cases) received TAE for severe bleeding after PCNL. Before TAE, the conservative therapy included drainage tube clamping, hemostatic drugs, antibiotics, adequate hydration, blood transfusion, and absolute bed rest. If bleeding persisted (continuing or intermittent hematuria that led to a further drop in hematocrit, tachycardia, and hypotension), selective renal arteriography with embolization was used to stop the bleeding. The successful hemostasis rate of conservative therapy was 73.6% (89/121) and that of TAE was 100% (32/32). The demographic and clinical data between the two groups were presented in Table 1. The mean platelet counts before PCNL had no difference between the two groups (*P*=0.228). No difference was found between the two groups about patients receiving anticoagulation or antiplatelet therapy. Univariate analysis indicated that advanced age (*P*=0.039), preoperative urinary tract infection (*P*=0.024), and diabetes mellitus needing embolization (*P*=0.001) were associated with severe postoperative bleeding. Stone size and types did not increase the risk of severe bleeding needing TAE (*P*=0.483 and* P*=0.170). The location of calyx entry had no difference between the two groups (*P*=0.246). There was a significantly higher blood loss for patients who received TAE than for patients who only received conservative therapy (58.3 vs. 15.3 g/l,* P*<0.001). In the TAE group, 56.3% of patients received transfusion, and the mean volume was 6.7 units, which were significantly higher than the conservative treatment group (56.3% vs. 22.5%,* P*<0.001; 6.7 vs. 2.7,* P*=0.002). And patients had prolonged hospitalization days in the TAE group compared with the control group (7.5 days vs. 4.1 days,* P*<0.001).

The potential risk factors, including age, stone size, stone type, diabetes mellitus, urinary tract infection, and puncture site, were entered into multivariate analysis to identify the independent factors for post-PCNL severe bleeding. Results of multivariate analysis were shown in Table 2. Multivariate logistic regression analysis identified that diabetes mellitus was independently associated with severe postbleeding requiring TAE (OR: 3.778; 95% CI: 1.276-11.190; and* P*=0.016).

**Figure 1:**
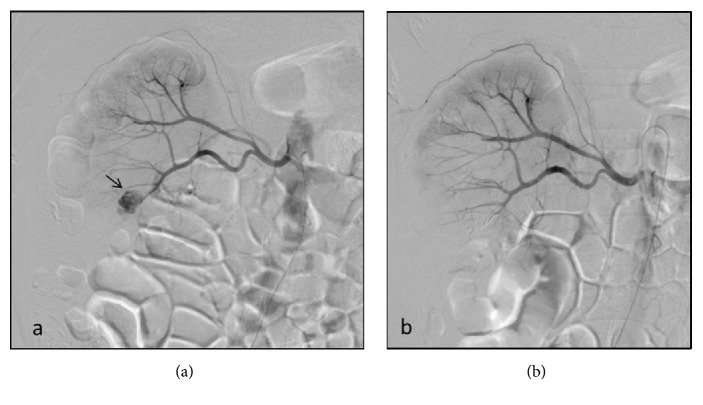
(a) Selective renal arteriography shows a PA arising from the right inferior anterior interlobar artery in a 49-year-old woman with renal hemorrhage 5 days after PCNL. (b) After finding the responsible vessel and embolized with NBCA and coils, angiographic imaging shows the disappearance of the PA.

**Figure 2:**
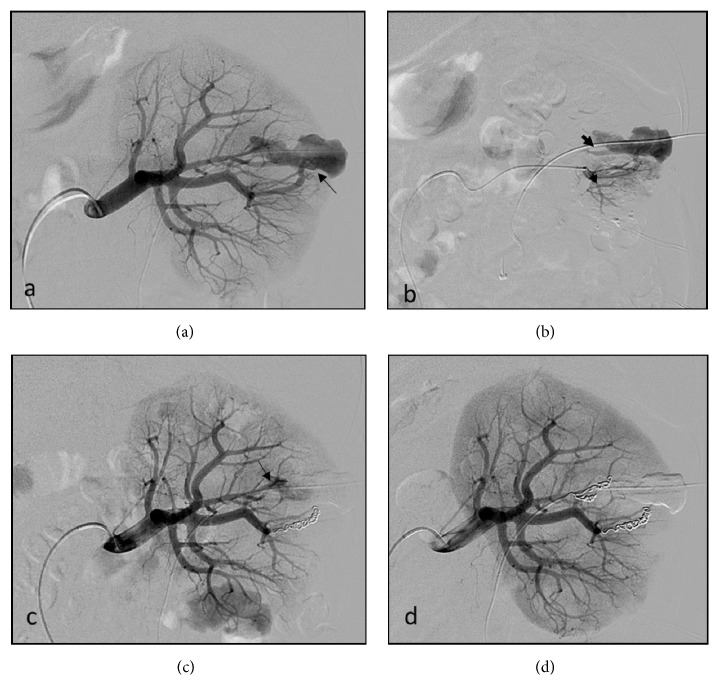
(a) The angiographic imaging in a 56-year-old man with renal hemorrhage 3 days after PCNL indicates a PA arise from the superior anterior interlobar artery (thin arrow). (b) Angiographic imaging show that an AVF (thick arrowhead) arise from the posterior and superior anterior interlobar artery. (c, d) Angiogram after coil embolization demonstrates complete occlusion of the PA and AVF.

**Figure 3:**
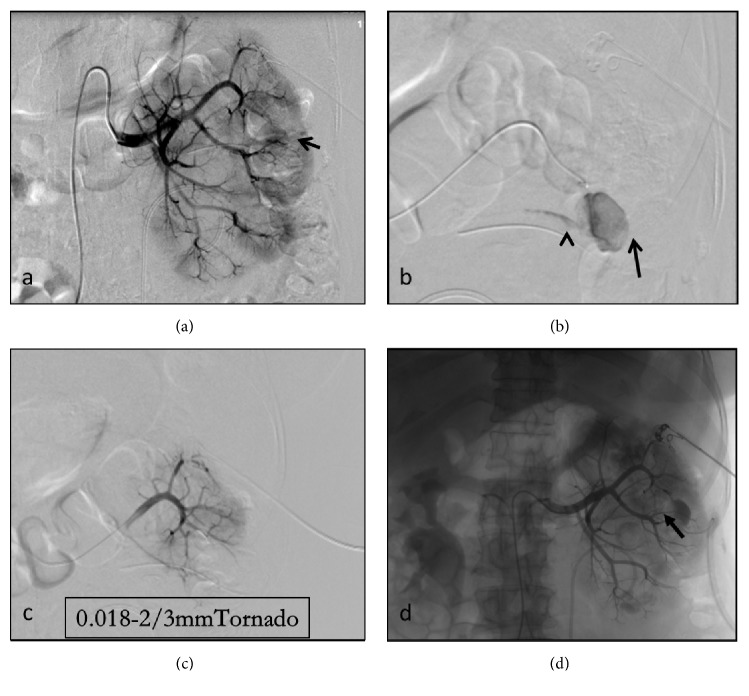
(a) Selective renal arteriogram shows a PA arise from the left posterior anterior segmental artery in a 54-year-old man. (b) Renal arteriography during the arterial phase shows a PA (thin arrow) and contrast agent extravasation along the drainage pipe (thick arrowhead) arising from the posterior and superior anterior interlobar artery. (c) After embolization with 3 coils (TORNADO MWCE-18S-3.0-2, Cook Inc.), there is no contrast medium extravasation again under angiogram. (d) After embolization, digital subtraction angiographic imaging shows complete occlusion of the PA and contrast agent retention.

**Figure 4:**
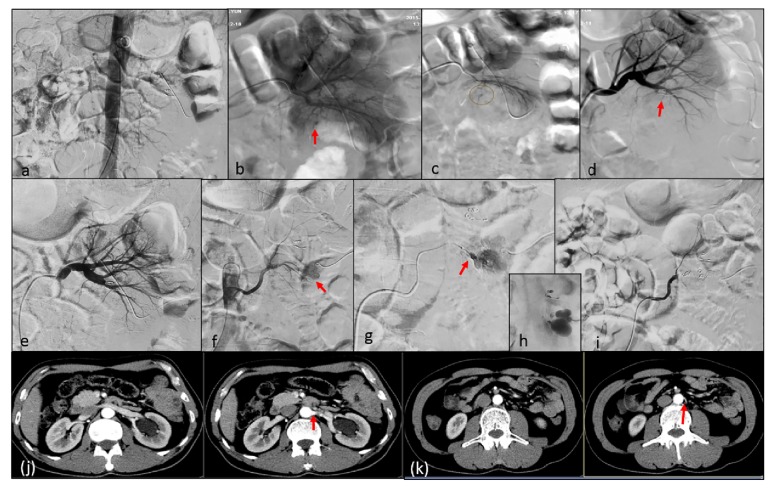
Secondary embolization in a patient with PA after PCNL. (a) Abdominal aorta angiography cannot show the number of left renal artery clearly because of the interference of intestinal gas. (b, c) Selective left renal arteriography shows a PA (red arrow). (d) Angiogram after coil embolization showing complete occlusion of the PA. (e-h) Angiogram 3-day after first time TAE shows a new PA (red arrow) arising from accessory left renal artery, which arises at approximately the inferior level of third Lumbar vertebra. (i) After coil embolization, angiogram shows complete occlusion of the PA. (j, k) The CT image shows the location of left renal artery and accessory left renal artery.

The operation information during TAE was summarized in Table 3. The angiographic imaging showed that 3 patients with vascular injuries located in upper renal pole, 7 patients with vascular injuries located in middle renal pole, 20 patients with vascular injuries located in in lower renal pole, and 2 patients with vascular injuries located in middle and lower renal pole. The following was shown: 14 patients with contrast medium extravasation, 10 patients with PA, 4 patients with AVF, and 4 patients with composite lesions. All of the patients had successfully stopped bleeding and some representative cases were depicted in Figures 1–4. The embolic material used in TAE was coil in 27 patients, coil and gelatin sponge in 4 patients, and coil combined with NBCA in 1 patient. The mean number of coils used was 3 (range, 1 to 12 coils). The angiogram was performed to evaluate the renal parenchymal loss, and there was no patient with parenchymal loss more than 25% of the total renal volume. One patient was successfully controlled by receiving a second TAE for rehemorrhage. Also, in the 8 patients with solitary or functional solitary kidney, 1 received TAE and 7 only received conservative treatment. They were all followed up intensively, and no further deterioration of renal function was observed. Endovascular embolization as distant as possible was performed in all 32 patients to reduce the loss of kidney parenchymal.

The mean time of follow-up was 18.6 months (range, 12 to 36 months) in all patients. No major complications of embolization (renal artery dissection, coil migration, postembolization syndrome, and loss of renal function) occurred in patients received TAE. The values of SCr and eGFR in different time points were shown in Table 4 (Patients with eGFR more than 120 ml/min/1.73m^2^ were recorded as 120 ml/min/1.73m^2^.) No statistical difference of SCr was observed between the two groups at preoperative PCNL and at last follow-up (0.95 vs. 0.91 mg/dl,* P*=0.446 and 0.95 vs. 0.92 mg/dl,* P*=0.635) and there was no significant difference in eGFR (86.77 vs. 88.87 ml/min/1.73m^2^,* P*=0.552 and 86.18 vs. 85.11,* P*=0.770). Moreover, no differences of SCr and eGFR were found before PCNL and at last follow-up in the TAE group (0.95 vs. 0.95 mg/dl,* P*=0.857 and 86.77 vs. 86.18 ml/min/1.73m^2^,* P*=0.715), and also no differences were found in the conservative treatment group (0.91 vs. 0.92 mg/dl,* P*=0.698 and 88.87 vs. 85.11 ml/min/1.73m^2^,* P*=0.125).

## 5. Discussion

PCNL is an efficient way for kidney stones and ureteral stones [15, 16]. Bleeding is one of the most common clinical complications of PCNL. In most patients, post-PCNL hemorrhage could be controlled by conservative therapy. However, TAE has been an established therapy for severe, persistent, or intermittent hemorrhage after PCNL that cannot be stopped by conservative treatment [17, 18]. In previous literatures, large stone burdens with multiple punctures, lower renal puncture site, stone number and types, history of ipsilateral renal stone surgery, and occurrence of intraoperative pelvicalyceal perforation have been demonstrated as the risk factors for severe post-PCNL hemorrhage [19–22]. Moreover, established studies showed that patients with tubeless drainage after PCNL were also associated with an increased risk of bleeding and subsequent angioembolization [22]. However, there were various data with regards to the risk factors of post-PCNL severe bleeding, which needs to be further confirmed. In this study, we compared patients who received TAE with patients who only received conservative therapy to evaluate the efficiency of endovascular embolization and the potential related risk factors.

In our study, the blood transfusion rate was 4.7% (38/812) for patients with post-PCNL hemorrhage. The embolization rate after PCNL in our hospital was 3.9% (32/812). In univariate analysis, advanced age, preoperative urinary tract infection, and diabetes mellitus needing embolization were shown to be associated with post-PCNL severe bleeding. For patients with advanced age, the ability of repairing injury declined, which could result increased blood losing. For preoperative urinary tract infection, the possible explanations included the hyperemic nature of inflamed urothelium, or distorted anatomy secondary to edema. However, advanced age and preoperative urinary tract infection did not reveal statistical significance in multivariate analysis. This may be explained by the small number of patients included in our study. Diabetes mellitus was an independent predictive factor affecting the bleeding risk, which agree with the study of Akman et al. and Kurtulus et al. [23, 24]. The relationship between diabetes and bleeding after the initial trauma of tract formation was explained with arteriosclerosis. In addition, diabetes affects the whole vascular system, resulting in microangiopathies, which are highly vulnerable to bleeding [25].

The stone size and types did not increase the risk of post-PCNL severe bleeding (*P*=0.483 and* P*=0.170). Also, the different puncture site location had no correlation with increased bleeding risk (*P*=0.246). Patients with history of urinary system operation were recorded as extracorporeal shock-wave lithotripsy (ESWL), PCNL, and previous ipsilateral open renal surgery (POS), and no evidence indicated increased risk of bleeding. The blood loss and transfusion units of patients who received TAE were obviously higher than patients who only received conservative therapy. Therefore, patients receiving TAE might suffer severe renal vascular injury and severe bleeding, and they might be more recommended to angiographic embolization.

Arterial lesions are rare but often ineffectively treated by conservative means. Owing to the high pressure, renal arterial bleeding can drain into renal parenchyma or hilar areolar tissue resulting in a PA [3, 26]. In accordance with previous studies, contrast medium extravasation and PA were the most common types of angiographic manifestations [10, 11, 27]. In previous study, the major limitations of arterial embolization with NBCA, gelatin sponge, PVA, and Embosphere include the reflux of the embolic agent to the normal artery, which leads to unwanted regional embolization; passing to the venous system from AVF, which leads to pulmonary embolism; passing to the collecting system from ACF, which leads to acute urinary obstruction; and inadequacy in occluding large vessels injury [28]. In our experience, coils, as a permanent embolic agent, were an effective renal arterial occlusion tool, particularly in patients with small artery lesions.

No complication of coil migration was found in long-term follow-up. In previous literature, the efficacy of TAE in treating post-PCNL hemorrhage was 88%-100% [7, 29]. However, ischemia in renal parenchyma is one problem of TAE that may affect the renal function [29]. Moreover, the long-time effects of contrast medium on renal function should be considered as well. In this study, the ultrasonography or CT imaging in postoperative follow-up showed that the shrinking of the infarct area and the development of collaterals were found in TAE patients. No recanalization or migration of coil was found in long-term follow-up. No patients with loss of parenchymal larger than 25% of the total renal volume were found, and renal functions were effectively reserved in long-term follow-up. This may owe to the effective compensation of contralateral normal kidney. In all, TAE was an effective method to resolve post-PCNL severe hemorrhage without obvious side effect on renal function.

From this study and our experience, we proposed 3 aspects that should be given special attention. First, TAE is a safe and effective procedure to control post-PCNL severe bleeding without deterioration of renal function, even for patients receiving repeated TAE. Second, patients, who complicated with persisted or intermittent hematuria requiring multiple blood transfusion, can receive early angiogram and TAE to reduce the hospitalization days and the unit of blood transfusion. Third, to avoid the risks of post-PCNL severe bleeding, surgeons should operate more prudently and carefully on patients with diabetes mellitus.

The limitations of the present study were its retrospective nature, which introduced selection biases. Even though TAE has been regarded as a standard method for severe bleeding after PCNL, there is no criteria consensus about how and when to intervene by interventional therapy. Hence, the ultimate decision to use TAE was made on a case-by-case basis by the operator, which introduced additional selection bias. Lack of standardized criteria to evaluate the loss of renal parenchymal introduces another potential bias.

## 6. Conclusions

Severe renal hemorrhage is a rare but serious complication of PCNL. TAE is a safe and effective minimally invasive treatment for renal hemorrhage after PCNL that cannot be controlled by conservative therapy. Early TAE may decrease the units of blood transfusion and hospitalization days for patients. Selective catheterization and embolization can help reserve renal function and reduce the potential risk of nephrectomy. Moreover, patients with diabetes mellitus are correlated with an increased risk of post-PCNL renal hemorrhage needing TAE.

## Figures and Tables

**Table 1:** Demographic and clinical difference between patients in the TAE group and conservative treatment group.

Characteristic	All	TAE group	Control group	*P* value
	(n=121)	(n=32)	(n=89)	
Age	51.9 ± 11.8	55.6 ± 9.6	50.6 ± 12.3	0.039*∗*
Gender (male)				0.649
Male	87(71.9)	24(75.0)	63(70.8)	
Female	34(28.1)	8(25.0)	26(29.2)	
Platelet count (×10^∧^9/L)	203.7 ± 53.4	193.7 ± 55.0	207.3 ± 52.6	0.228
Stone size				0.483
<2	44(36.4)	10(31.3)	34(38.2)	
≥2 cm	77(63.6)	22(68.7)	55(61.8)	
Stone type				0.170
Solitary	34(28.1)	6(18.8)	28(31.5)	
Multiple or Staghorn	87(71.9)	26(81.2)	61(68.5)	
Number of tracts				0.394
Simple	116(95.9)	32(100.0)	84(94.4)	
Multiple	5(4.1)	0(0)	5(5.6)	
Urinary tract infection				0.024*∗*
Yes	55(45.5)	20(62.5)	35(39.3)	
No	66(54.5)	12(37.5)	54(60.7)	
Hypertension				0.436
Yes	46(38.0)	14(43.8)	32(36.0)	
No	75(62.0)	18(56.2)	57(64.0)	
Diabetes mellitus				0.001*∗*
Yes	22(18.2)	12(37.5)	10(11.2)	
No	99(81.8)	20(62.5)	79(88.8)	
Antiplatelet therapy				0.567
Yes	7(5.8)	3(9.4)	4(4.5)	
No	114(94.2)	29(90.6)	85(95.5)	
Anticoagulation therapy				1.000
Yes	4(3.3)	1(3.1)	3(3.4)	
No	117(96.7)	31(96.9)	86(96.6)	
History of operation				0.831
None	67(55.4)	18(56.3)	49(55.1)	
ESWL	13(10.7)	4(12.5)	9(10.1)	
PCNL and/or POS	41(33.9)	10(31.2)	31(34.8)	
Operation site				0.260
Right	54(44.6)	17(53.1)	37(41.6)	
Left	67(55.4)	15(46.9)	52(58.4)	
Puncture site				0.246
Upper calyx	17(14.0)	5(15.6)	12(13.5)	
Middle calyx	64(52.9)	13(40.6)	51(57.3)	
Inferior calyx	40(33.1)	14(43.8)	26(29.2)	
Hemoglobin decrease (g/L)	26.4 ± 24.6	58.3 ± 18.5	15.3 ± 14.7	<0.001*∗*
Numbers of patients receiving transfusion				<0.001*∗*
Yes	38(31.4)	18(56.3)	20(22.5)	
No	83(68.6)	14(43.8)	69(77.5)	
Units of transfusion		6.7 ± 4.4	2.7 ± 1.5	0.001*∗*
Duration of hospitalization (days)		7.5 ± 4.1	4.2 ± 1.5	<0.001*∗*

ESWL: extracorporeal shock wave lithotripsy; PCNL: percutaneous nephrolithotomy; POS: previous ipsilateral open renal surgery. *∗P*<0.05.

**Table 2:** Multivariate logistic analysis of the potential factors for severe bleeding needing TAE.

Factors	Characteristics		OR	95% CI	*P* values
	Unfavorable	Favorable			
Age			1.027	0.984-1.073	0.224
Stone size	≥2cm	<2cm	1.276	0.471-3.452	0.632
Stone type	Multiple or Staghorn	Solitary	2.614	0.825-8.276	0.102
Urinary tract infection	Yes	No	2.063	0.806-5.283	0.131
Diabetes mellitus	Yes	No	3.778	1.276-11.190	0.016*∗*
Puncture site	Inferior calyx	Middle or upper calyx	1.905	0.742-4.893	0.180

OR: odds ratio; CI: confidence interval. *∗P*<0.05.

**Table 3:** Operation information about TAE groups.

Characteristic	Number	Percent (%)
Site of vascular lesions (n)		
Upper pole	3	9.4
Middle pole	7	21.9
Lower pole	20	62.5
Middle & lower pole	2	6.2
Angiographic findings (n)		
Contrast extravasation	14	43.7
PA	10	31.3
AVF	4	12.5
PA & AVF	2	6.3
PA & ACF	1	3.1
PA & AVF & AVF	1	3.1
Embolic material used (n)		
Coil	27	84.4
Coil & GS	4	12.5
Coil & Glue	1	3.1
Parenchymal loss (%)		
Less than 25%	32	100

PA: pseudoaneurysm; AVF: renal arteriovenous fistula; ACF: arteriocalyceal fistula; and GS: gelatin sponge.

**Table 4:** The comparison of laboratory examination data perioperative and long-term renal function outcomes between groups.

Variable	TAE group	Control group	*P* values
	(n=32)	(n=89)	
Preoperative renal function			
SCr (mg/dl)	0.95 ± 0.26	0.91 ± 0.23	0.446
eGFR (ml/min/1.73m^2^)	86.77 ± 18.76	88.87 ± 15.99	0.552
Postoperative renal function			
SCr	0.94 ± 0.25	0.93 ± 0.22	0.761
eGFR (ml/min/1.73m^2^)	87.60 ± 20.24	86.41 ± 18.95	0.775
Renal function at lost follow-up			
SCr (mg/dl)	0.95 ± 0.30	0.92 ± 0.28	0.635
eGFR (ml/min/1.73m^2^)	86.18 ± 15.78	85.11 ± 16.95	0.770
Renal function change between preoperational and at lost follow-up			
SCr (*P* value)	0.857	0.698	
eGFR (*P* value)	0.715	0.125	

eGFR: estimated glomerular filtration rate; SCr: serum creatinine; and TAE: transcatheter arterial embolization. *∗P*<*0.05*.

## Data Availability

The data used to support the findings of this study are available from the corresponding author upon appropriate request.

## Abbreviations

PCNL: Percutaneous nephrolithotomy
TAE: Transcatheter arterial embolization
eGFR: Estimated glomerular filtration rate
PA: Pseudoaneurysm
AVF: Arteriovenous fistula
ACF: Arteriocalyceal fistula
CT: Computed tomography
HB: Hemoglobin
CRP: C-reactive protein
SCr: Serum creatinine
DSA: Digital subtraction angiography
NBCA: * n*-Butyl-2-cyanoacrylate
ESWL: Extracorporeal shock-wave lithotripsy
OR: Odds ratio
CI: Confidence interval.
